# Supplementary material for: Use of Antibiotics and Probiotics Reduces the Risk of Metachronous Gastric Cancer after Endoscopic Resection
Source: Biology (Basel). 2021 May 22;10(6):455. doi: 10.3390/biology10060455 (PMC8224738; doi:10.3390/biology10060455)
Supplement: Supplementary file 1 [file biology-10-00455-s001.zip › supplementary 1.pdf]

Supplementary Table 1. ICD-10 codes for comorbidities.

Comorbidity	ICD-10 codes
Atrial fibrillation	I480–I489
AIDS	B200–B229, B24
Arterial thrombosis	I740–I749
Carotid disease	I652, I720
Cerebrovascular disease	G450-G469, H340, I600–639, I64, I650–I699
Chronic heart failure	I099, I110, I130, I132, I255, I420, I425–I439, I500–I509, P290
Chronic kidney disease < stage 5	I120, I131, N032–N037, N052–N057, N180–N189, N19, N250, Z490–Z492, Z940, Z992
Chronic kidney disease stage 5	N185
Dementia	F000–F029, F03, F051, G300–G309, G311
DM without complication	E100, E101, E106, E108–E111, E116, E118–E121, E126, E128–E131, E136, E138–E141, E146, E148, E149
DM with complications	E102–E105, E107, E112–E117, E122–E125, E132–E135, E137, E142–E145, E147
Deep vein thrombosis	I800–I809, I820–I829
Hemiplegia	G041, G114, G801, G802, G810–G834, G839
Hypertension	I10, I110–I159
Dyslipidemia	E780–E785
Ischemic heart disease	I210–I229, I252
Liver disorder (mild)	B180–B189, K700–K703, K709, K713–K715, K717, K730–K749, K760, K762–K764, K768–K769, Z944
Liver disorder (severe)	I850, I859, I864, I982, K704, K711, K721, K729, K765–K767
Malignancy without metastasis	C000–C009, C01, C020–C69, C07, C080–C119, C12, C130–C189, C19, C20, C210–C229, C23, C240–C329, C33, C340–C349, C37, C380–C519, C52, C530–C549, C55,C56, C570–C570, C58, C600–C609, C61, C620–C639, C64, C65, C66, C670–C729, C73, C740–C769, C810–C969, C97
Malignancy with metastasis	C770–C809
Pulmonary embolism	I260–I269
Peripheral vascular disease	I700–I719, I731, I738, I739, I771, I790, I792, K551, K558, K559, Z958, Z959
Pulmonary disease	I278, I279, J40, J410–J419, J42, J430–J459, J46, J47, J60, J61, J620–J639, J64, J65, J660–J679, J684, J701, J703
Rheumatic disease	M050–M069, M315, M320–M349, M351, M353, M360
Transient ischemic attack	G459
Peptic ulcer disease	K250–K289
Unstable angina	I200–I209
Valvular disease	I340–I379

Abbreviations: AIDS, acquired immunodeficiency syndrome; DM, diabetes mellitus.

Supplementary Figure 1. Hazard ratios for metachronous gastric cancer based on clinical factors after endoscopic resection.

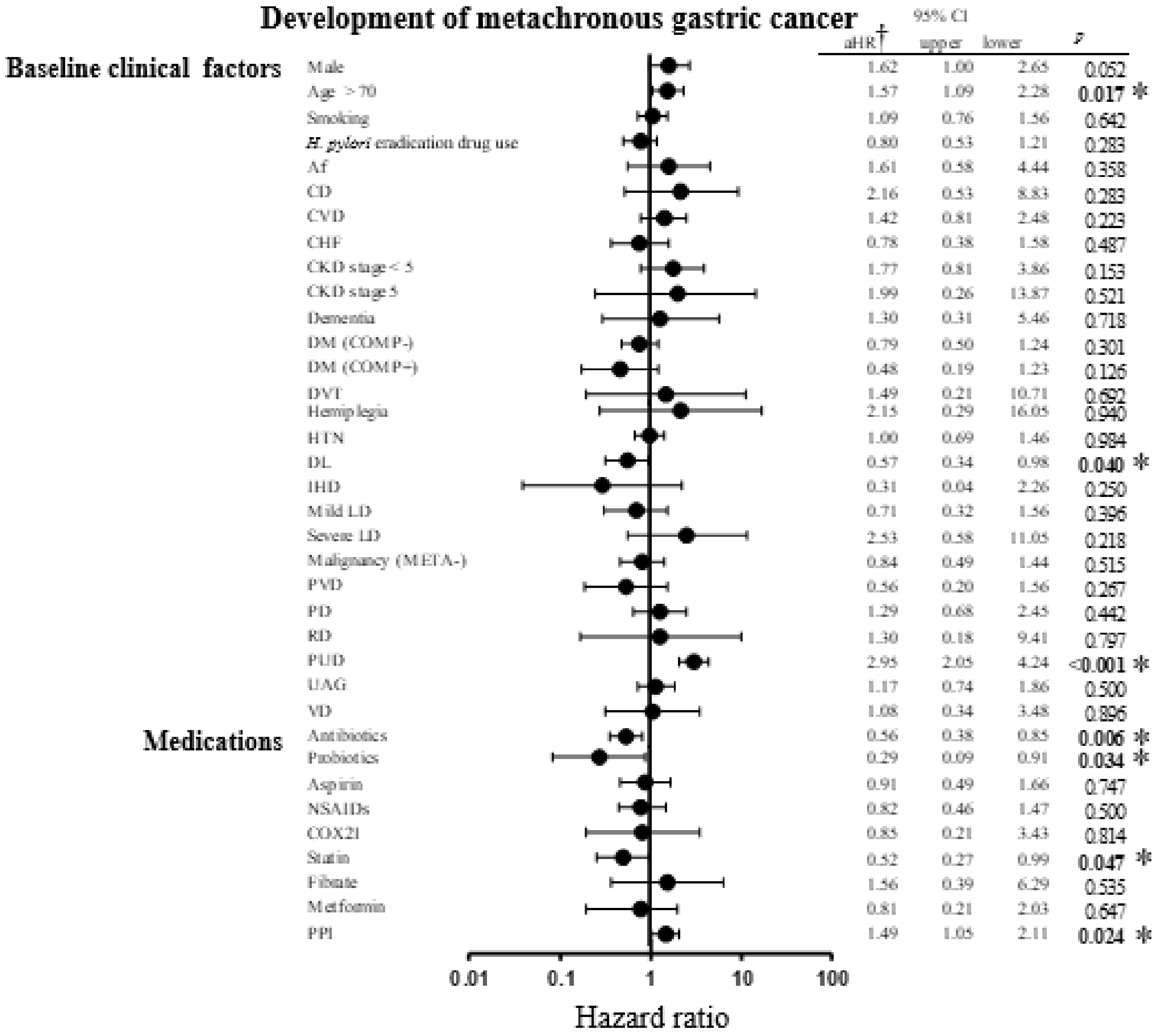

Abbreviations: CI, confidence interval; aHR, adjusted hazard ratio; Af, atrial fibrillation; CD, carotid disease; CVD, cerebrovascular disease; CHF, chronic heart failure; CKD, chronic kidney disease; DM, diabetes mellitus; COMP, complications; DVT, deep vein thrombosis; HTN, hypertension; DL, dyslipidemia; IHD, ischemic heart disease; LD, liver disorder; META, metastasis; PVD, peripheral vascular disease; PD, pulmonary disease; RD, rheumatic disease; PUD, peptic ulcer disease; UAG, unstable angina; VD, valvular disease; NSAID, nonsteroidal anti-inflammatory drug; COX2I, cyclooxygenase-2 inhibitor; PPI, proton pump inhibitor.

† HR adjusted for age > 70 years, sex, smoking, and Charlson Comorbidity Index.

Supplementary Figure 2. Interactions of (A) antibiotics and (B) probiotics with all other medications.

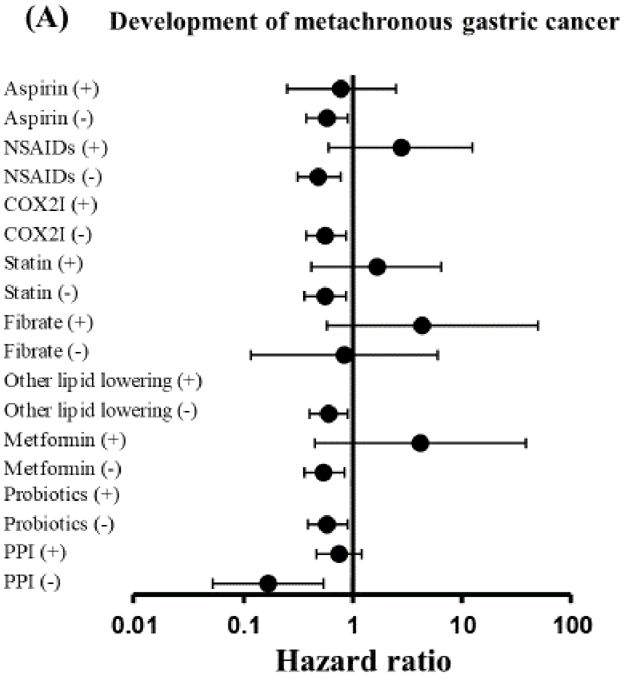

	aHR	95% CI upper	95% CI lower
Aspirin (+)	0.78	0.25	2.47
Aspirin (-)	0.57	0.37	0.88
NSAIDs (+)	2.73	0.60	12.46
NSAIDs (-)	0.49	0.31	0.78
COX2I (+)	NA	NA	NA
COX2I (-)	0.57	0.38	0.86
Statin (+)	1.64	0.42	6.43
Statin (-)	0.57	0.37	0.87
Fibrate (+)	4.35	0.58	49.56
Fibrate (-)	0.83	0.12	5.95
Other lipid lowering (+)	NA	NA	NA
Other lipid lowering (-)	0.59	0.40	0.89
Metformin (+)	4.18	0.46	38.29
Metformin (-)	0.54	0.36	0.83
Probiotics (+)	NA	NA	NA
Probiotics (-)	0.59	0.39	0.91
PPI (+)	0.76	0.47	1.21
PPI (-)	0.17	0.05	0.54

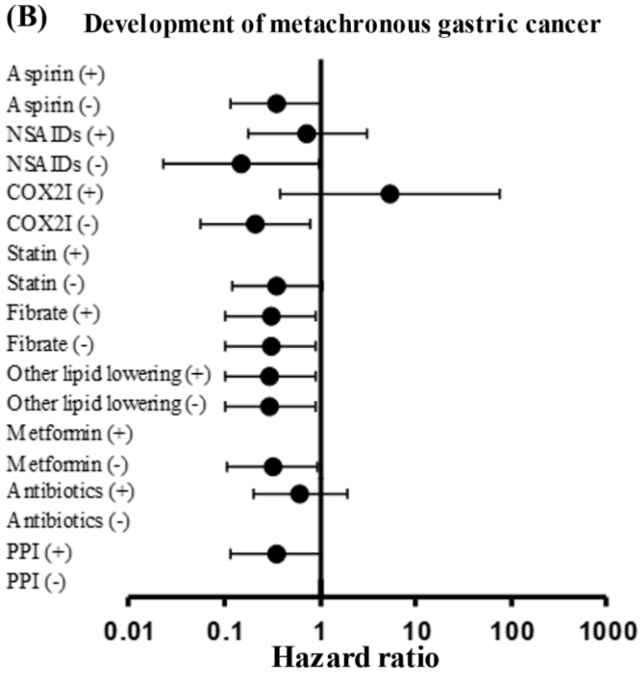

	aHR †	95% CI upper	95% CI lower
Aspirin (+)	NA	NA	NA
Aspirin (-)	0.32	0.10	1.03
NSAIDs (+)	0.72	0.16	3.25
NSAIDs (-)	0.13	0.02	0.96
COX2I (+)	6.04	0.36	101.75
COX2I (-)	0.19	0.05	0.79
Statin (+)	NA	NA	NA
Statin (-)	0.33	0.10	1.05
Fibrate (+)	0.28	0.09	0.90
Fibrate (-)	0.28	0.09	0.90
Other lipid lowering (+)	0.28	0.09	0.88
Other lipid lowering (-)	0.28	0.09	0.88
Metformin (+)	NA	NA	NA
Metformin (-)	0.29	0.09	0.94
Antibiotics (+)	0.60	0.18	2.01
Antibiotics (-)	NA	NA	NA
PPI (+)	0.32	0.10	1.02
PPI (-)	NA	NA	NA

Abbreviations: CI, confidence interval; aHR, adjusted hazard ratio; NSAID, nonsteroidal anti-inflammatory drug; COX2I, cyclooxygenase-2 inhibitor; PPI, proton pump inhibitor.
† HR adjusted for age > 70 years, sex, smoking, eradication of *H. pylori*, and Charlson Comorbidity Index.

Supplementary Figure 3. Cumulative incidence of metachronous gastric cancer in antibiotic users vs. non-users in propensity score matched cohort, calculated by age, sex, charlson comorbidity index, and several infections.

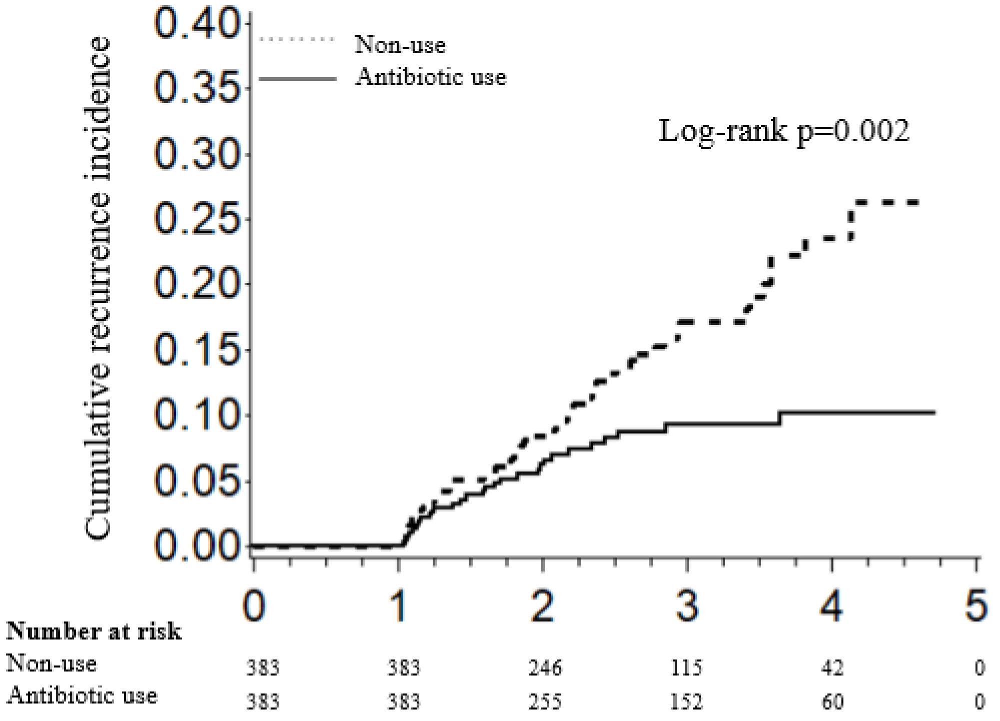

Survival analysis was performed using the Kaplan–Meier method and log-rank test.
