# Supplementary material for: Use of Antibiotics and Probiotics Reduces the Risk of Metachronous Gastric Cancer after Endoscopic Resection
Source: Biology (Basel). 2021 May 22;10(6):455. doi: 10.3390/biology10060455 (PMC8224738; doi:10.3390/biology10060455)
Supplement: Supplementary file 1 [file biology-10-00455-s001.zip › Supplementary table2.pdf]

Supplementary Table2. Drug codes.

Antibiotics
620008598
620008599
622224801
621926801
610451000
622461001
622588701
620006791
620008047
620009408
621731302
621738802
622745000
616130110
616130112
616130469
621086806
621087001
621087903
621088403
621088705
621088802
621088901
621089103
621089501
621089701
622316800
622476500
622612400
620003645
620003646
620003647
620004958
610411057
610411058
610411059
620009331
620009332
621930801
621930901
621931001
621940201
621940301
621940401
621942904
621943004
621943104
621946301
621947501
621947601
621955901
621956001
621956101
621962401
621962501
621962601
621962701
620004080
620008746
620005499
620005500
620005501
620005502
620005503
620005505
620005506
620005507
620005508
622559401
622644301
622745800
622745900
610454083
616150063
616150088
620007109
621124301
621124604
621124801
621125101
621125401
621125701
621126004
622746300
622746400
622746500
621123301
621148101
621148901
621148201
621149001
620006753
622612300
621925701
621925801
621925901
621925701
621925801
621925901
622365901
622366001
622367801
622367901
622368201
622368301
622369001
622369101
622370801
622370901
622371001
622372501
622372601
622372701
622372801
622372901
622376301

622376401	622400701	621072701
622377901	622400801	621073704
622378001	622400901	621073901
622379101	622401001	622066501
622379201	622435301	622066502
622380001	622435401	622066601
622380101	622436801	622066602
622380301	622436901	622111101
622380401	622476600	622127801
622382001	622476700	622165902
622382101	621489001	622745100
622383001	621489102	622745200
622383101	621489203	622745300
622383501	621489402	622745400
622383601	621489502	616130332
622384801	621489601	616130333
622384901	621946502	616130512
622386101	621964702	616130513
622386201	621976900	621113002
622386801	622096202	621113712
622386901	622125902	621964002
622387801	622311500	610443024
622387901	620006026	610443026
622388801	620006027	610451034
622388901	621122501	620009094
622389401	621122601	621348401
622389501	620007057	622269401
622391101	621116201	622269501
622391201	621116301	622269601
622391701	616130132	622270801
622391801	620006919	622270901
622392301	620006920	622274201
622392401	622054901	622274301
622394001	616130295	622274401
622394101	620007024	622274501
622394401	620007025	622274601
622394501	620007026	622275601
622395201	620005906	622276801
622395301	620006829	622281501
622395901	620008584	622281601
622396001	620009117	622282801

	622286701
	622290701
	622290801
	622294801
	622295301
	622295401
	622296901
	622352901
	622353001
	622353101
	622368001
	622411501
	616140105
	620003926
	620003927
	620003928
	620003929
	620003930
	620003931
	620003932
	620003933
	620003934
	620003935
	620003939
	620006670
	620008013
	621736701
	621742103
	621752901
	622079401
	622659101
	622746200
	616140102
	610411055
	610411056
	620008702
Probiotics	
	612370052
	612370059
	612370066
	612370067
	620000094
	620003183
	620006873
	620008578
	620008690
	620008698
	620418104
	620418301
	620418401
	620418501
	620420001
	620420101
	620420301
	620421101
	620421201
	620421401
	620421501
	621270001
	622113001
	622451201
	622451301
	622464601
Combination agents of pylori eradication	
	610462048
	610462049
	622029101
	622289101
	622289201
	622289301
	622485401
	622485501
	622485601
Amoxicillin	
	620006829
	620008584
	620009117
	621073901
	622745400
	616130040
	616130132
	620006919
	616130295
	620007025
Clarithromycin	
	616140105
	620003926
	620003927
	620003928
	620003929
	620003930
	620003931
	620003932
	620003933
	620003934
	620003935
	620003939
	620006670
	620008013
	621736701
	621742103
	621752901
	622079401
	622659101
	622746200
	616140102
Metronidazole	
	620007057
Proton Pump Inhibitors	
	610412202
	610412203
	610443068
	610443069
	610443070
	610443071
	610462010
	610462011
	612320549
	612320550
	620001983
	620001984

620001985
620001986
620002694
620002695
620002743
620002744
620002749
620002750
620002871
620002872
620003914
620004087
620004088
620005581
620005583
620005584
620005585
620007127
620007128
620009451
620009452
621622303
621622403
621630104
621630502
621630601
621630701
621673701
621673801
621680901
621681001
621681401
621693101
621693201
621743701
621743801
621780301
621780303
621780401
621780403
621794301
621796301
621796401
621919001
621919101
621977902
621983103
621997201
621997301
621999501
621999601
621999901
622000001
622001201
622001301
622002201
622002301
622005301
622005401
622007301
622007401
622011201
622011301
622012901
622013001
622015801
622015901
622016201
622016301
622020602
622020702
622021401
622021501
622023101
622023201
622023301
622023401
622025601
622025701
622026001
622026101
622031601
622031701
622032801
622032901
622034601
622034701
622035501
622035601
622040101
622040201
622060201
622060301
622077801
622080701
622080801
622089501
622089601
622118601
622118701
622402601
622469801
622469901
622505501
622505601
622617000
622617100
622617200
622624801
622624901

Potassium Competitive Acid Blockers

622404401
622404501

Non-Steroidal Anti-Inflammatory Drugs

610406382
610406383
610406384
610406387
610406388
610406402
610422322

610433119
610443079
610443080
610454052
610463033
610463034
610463037
610463150
611140098
611140138
611140139
611140236
611140237
611140322
611140323
611140395
611140431
611140435
611140828
611140844
611140845
611140846
611140847
620002043
620002057
620002431
620002432
620002516
620002531
620002537
620002646
620002647
620003153
620003154
620003523
620003524
620003624
620004494
620004626
620004857
620004858
620004916
620004917
620006095
620006174
620006848
620006849
620006859
620007059
620007068
620007095
620007096
620007098
620007099
620007100
620007129
620007150
620007151
620007152
620007153
620008114
620008115
620008117
620008118
620008119
620008120
620008121
620008122
620008123
620008124
620008125
620008126
620008127
620008128
620008129
620008130
620008131
620008132
620008133
620008135
620008136
620008137
620008138
620008139
620008140
620008141
620008142
620008143
620008144
620008145
620008146
620008147
620008148
620008149
620008150
620008151
620008625
620008628
620008632
620008646
620008780
620079303
620079305
620079311
620079315
620079325
620079338
620079345
620081301
620088902
620090601
620094401
620097508
620097815
620098401
620098501
620098702
620098801
620098902
620099003
620099101
620099201
620099301

	620099501
	620099601
	620099701
	620100001
	620100501
	620100602
	620100702
	620100901
	621212601
	621215101
	621215401
	621215602
	621392002
	621466002
	621466202
	621466401
	621466601
	621534501
	621623201
	621634301
	621635802
	621640201
	621640501
	621808201
	621837703
	621837803
	621936001
	621981502
	622011102
	622012401
	622014601
	622022501
	622034902
	622051201
	622058201
	622062601
	622066701
	622314000
	622325600
Aspirin	
	610443053
	611140017
	611140798
	611140849
	611140850
	620000065
	620000484
	620001952
	620004280
	620007816
	620008577
	620009301
	620072734
	621362001
	621374801
	621374901
	621375001
	621391201
	621419201
	621419401
	621675501
	621676502
	622258001
Statin	
	610443013
	610443014
	610454084
	610454085
	610462015
	610462016
	610470012
	610470013
	610470014
	612180263
	612180264
	612180265
	620000052
	620000053
	620000103
	620000104
	620000105
	620000106
	620000107
	620000108
	620000159
	620000160
	620000176
	620000422
	620000423
	620002477
	620002478
	620002736
	620002798
	620002799
	620002800
	620004038
	620008053
	620008054
	620008055
	620008056
	620009322
	620009323
	620009324
	620009325
	621521301
	621521401
	621523101
	621523201
	621524102
	621524402
	621525701
	621525801
	621528602
	621528702
	621528801
	621528901
	621529001
	621529101
	621531001
	621531101
	621531703
	621532501
	621532601

621532902	622052801	622186701
621533002	622055602	622187601
621533101	622071601	622187701
621533201	622075801	622204801
621533501	622075901	622204901
621533601	622076401	622217101
621533801	622076501	622217201
621533901	622098401	622239201
621534003	622098501	622239301
621534101	622099101	622241301
621534204	622099201	622241401
621534301	622102502	622244801
621623603	622107601	622244901
621635202	622107701	622252001
621639001	622110401	622252101
621639101	622110501	622268001
621639701	622116802	622268101
621639801	622116902	622268201
621643301	622126901	622269101
621643401	622127001	622269201
621643501	622128201	622270001
621643601	622128301	622270101
621675101	622136401	622271801
621694001	622139600	622271901
621752501	622143801	622273101
621934801	622143901	622273201
621934901	622152001	622273301
621935001	622152101	622274901
621948701	622161801	622275001
621948801	622161901	622275101
621955003	622165601	622276301
621964101	622165701	622276401
621964201	622167601	622276501
621964301	622167701	622280201
621964401	622169902	622280301
621964501	622170002	622280401
621964601	622170101	622280501
621981403	622170201	622280601
622015101	622180602	622280701
622015201	622180702	622280801
622015301	622186601	622282201

622282301	622315500	622524601
622283701	622321900	622524701
622283801	622342801	622528901
622285001	622347401	622529001
622285101	622359101	622537301
622286201	622360101	622537401
622286301	622362701	622568601
622286401	622365801	622571801
622287601	622372401	622571901
622289501	622387601	622572801
622289601	622392501	622572901
622291801	622406901	622575201
622291901	622419701	622575301
622292001	622419801	622575401
622292301	622419901	622575501
622292401	622421601	622575601
622292501	622421701	622575701
622293301	622421801	622577901
622293401	622426201	622578001
622294301	622427701	622578101
622294401	622427801	622578201
622294501	622431401	622578401
622296001	622434301	622578501
622296101	622434401	622578601
622296201	622441101	622578701
622297101	622441201	622578801
622297201	622457701	622581601
622298001	622457801	622581701
622298101	622457901	622581801
622298201	622464901	622581901
622299001	622465001	622582001
622299101	622465101	622582101
622302401	622475000	622582501
622302501	622475100	622582601
622302801	622512001	622582701
622302901	622512101	622582801
622304601	622512201	622584201
622304701	622522101	622584701
622304801	622522201	622584801
622304901	622522301	622586001
622315400	622524501	622586101

622586201
622586301
622588801
622588901
622589001
622589101
622590101
622590201
622590301
622590401
622591701
622591801
622591901
622592001
622592601
622592701
622592901
622593001
622593101
622593201
622595301
622595401
622598301
622598401
622598501
622598601
622599201
622599301
622599401
622599501
622600301
622600601
622600701
622600801
622600901
622601201
622601301
622601401
622601501
622604001
622604101
622604201
622605001
622605101
622605201
622605301
622605401
622606601
622606701
622615600
622615700
622615800
622615900
622640801
622640901
622644801
622644901
622660001
622660101
622665901
622666001
622666101
622676701
622676801
622691000
622691800
622692400
622692500
622692600
622692700
Fibrate
610407028
610422262
610422263
610422264
610422265
610422276
612180028
612180029
612180106
620002123
620008508
620338317
620339201
620339401
620339501
620340201
620340603
620340901
620341001
620341301
621254601
622026702
622039501
622039601
622090701
622090801
622096102
622096801
622096901
622223601
622573101
622590501
622590601
Other lipid lowering
610432003
610462007
610463087
612180004
612180140
612180141
612180292
620002508
620003669
620004459
620004868
620005785
620005920
620006115
620006870
620008631
620346008
620346018

	620346023	622654501
	620346029	
	620346101	
	622198801	
	622516701	
	622516801	
	622516901	
	622584201	
	622584701	
	622676701	
	622676801	
Metformin		
	620004480	
	620005570	
	621676001	
	621974701	
	621986301	
	621986401	
	622242501	
	622412701	
	622417101	
	622417201	
	622421101	
	622421201	
	622421901	
	622422001	
	622424401	
	622424501	
	622427201	
	622427301	
	622432601	
	622432701	
	622436301	
	622438401	
	622438501	
	622448601	
	622450301	
	622450401	
	622466601	
	622517101	
	622654401	
